# A novel, rapidly acquired and persistent spatial memory task that induces immediate early gene expression

**DOI:** 10.1186/1744-9081-6-35

**Published:** 2010-07-02

**Authors:** Lisa A Feldman, Matthew L Shapiro, Josephine Nalbantoglu

**Affiliations:** 1 Department of Neurology and Neurosurgery, McGill University and Montreal Neurological Institute, Montreal, Quebec H3A 2B4, Canada; 2 Neurobiology of Aging Laboratories, Research Center for Neurobiology, Mount Sinai School of Medicine, New York, NY 10029-6574, USA

## Abstract

**Background:**

The Morris water maze task is a hippocampus-dependent learning and memory test that typically takes between 3 days to 2 weeks of training. This task is used to assess spatial learning and induces the expression of genes known to be crucial to learning and memory in the hippocampus. A major caveat in the protocol is the prolonged duration of training, and difficulty of assessing the time during training in which animals have learned the task. We introduce here a condensed version of the task that like traditional water maze tasks, creates lasting hippocampus-dependent spatial cognitive maps and elicits gene expression following learning.

**Methods:**

This paradigm was designed for rats to quickly acquire a hippocampus-dependent spatial cognitive map and retain this memory for at least 24 hours. To accomplish this, we interspersed visible and hidden training trials, delivering them in a massed fashion so training takes a maximum of 15 minutes. Learning was assessed based on latencies to the platform during each training trial, as well as time spent in the goal quadrant during probe testing 30 minutes and 24 hours after training. Normal rats were compared to two impaired cohorts (rats with fimbria-fornix lesions and rats administered NMDA receptor antagonist (CPP)). To quantitate hippocampal expression of known learning genes, real-time polymerase chain reaction (RT-PCR) was performed on hippocampal cDNA.

**Results:**

We show that massed training using alternating visible and hidden training trials generates robust short-term working and long-term reference memories in rats. Like the traditional Morris water maze paradigm, this task requires proper hippocampal function, as rats with fimbria-fornix lesions and rats administered CPP fail to learn the spatial component of the task. Furthermore, training in this paradigm elicits hippocampal expression of genes upregulated following learning in a variety of spatial tasks: *homer1a, cfos *and *zif268*.

**Conclusions:**

We introduce here a condensed version of the Morris water maze, which is like a traditional water maze paradigm, in that it is hippocampus-dependent, and elicits hippocampal expression of learning genes. However, this task is administered in 15 minutes and induces spatial memory for at least 24 hours.

## Background

The Morris water maze is a spatial cognitive task that requires the creation of a hippocampus-dependent cognitive map of the environment. While the water maze is commonly used to differentiate learning between various cohorts of rodents, there are a number of disadvantages that limit the practicality of this task including the time required to sufficiently train animals, difficulty controlling for motivational or physical disabilities, and controlling for animal anxiety. There are further caveats that lie in the interpretation of water maze data including identifying when learning has taken place, and how to distinguish simple motor response learning from true spatial learning. We introduce here a novel abbreviated version of the water maze that was designed to overcome some of these limitations, to create a hippocampus-dependent spatial memory that persists for at least 24 hours, and which elicits gene expression of learning-related genes in the hippocampus.

Rodents are challenged in the Morris water maze to integrate environmental spatial cues and use them to locate a hidden platform in a pool of opaque water [1], thereby creating a spatial cognitive map of their environment. Animals are motivated to escape cool water by finding and climbing onto the hidden platform, thus the platform serves as the positive reinforcement in the task [2]. The training and testing schedules vary greatly across research institutions, however the general training protocol involves pre-training (which familiarizes the animal with the testing environment) the day prior to training, followed by a series of a few training trials per day over a period of 1-2 weeks, or multiple trials massed per day for 2-4 days. Memory is then assessed by a probe test that usually gives the animal 60 seconds to swim in the pool in which the hidden platform has been removed. Animals that have learned the location of the platform during training have shorter latencies to that quadrant, and spend more time in that goal quadrant as compared to other pool quadrants during the probe test [2]. As such, training and testing typically takes a minimum of three days, a relatively long time period in which it is difficult to assess when learning has occurred.

The duration of memory for training tasks is dependent on the number of training trials and the amount of time allotted between trials, whereby the longer inter-trial interval results in improved memory [3-5]. With each training trial, spatial information is learned and integrated into a cognitive map of the room, which is used to reduce latencies to the platform on further training trials and on the probe test. The temporal spacing of training trials for this task is imperative to the quality of spatial learning in rats [3] and mice [4]. Therefore, the manipulation of the number of training trials given per day, as well as the temporal spacing between trials is in critical balance for successful spatial learning.

Success in spatial tasks depends on proper hippocampal function, and as such, performance in the Morris water maze is especially sensitive to hippocampal damage. Both animals with hippocampal lesions [6], and animals with an intact hippocampus, but with lesions made to the fimbria-fornix [7-10] are dramatically impaired at both learning and memory performance in the task. Furthermore, hippocampal NMDA receptor participation is required for proper hippocampal learning as application of drugs that block these receptors, also block spatial learning [11-13]. Thus, spatial learning relies not only on an intact hippocampus, but on a hippocampus with intact afferent and efferent communication.

Among the numerous genes known to be upregulated in the hippocampus following spatial learning, members of the immediate early gene (IEG) subfamily have been extensively studied following maze learning. *C-fos *is a transcription factor whose upregulation correlates with spatial and behavior learning and memory in mice [14-19] and rats [16,17]. *Homer1a*, an effector IEG that regulates intracellular trafficking [20] and alters synapse formation [21,22], is upregulated following synaptogenesis and following training in multiple learning and memory models [23-25]. *Zif268 (Egr1, Krox24, NGFI-A) *expression is increased following water maze learning [26], as well as following learning [27] and memory [28] of hippocampus- and amygdala-dependent contextual fear conditioning tasks.

In this study we set out to design a spatial task that is based on the original Morris water maze but rapidly trains animals in a hippocampus-dependent fashion. Unlike classical versions of the task, this novel spatial paradigm is administered in 15 minutes of training trials. Our results demonstrate that successful acquisition in this paradigm elicits at least 24-hour long-term memory retention for spatial information. We show that successful learning of the task requires an intact hippocampus, as animals with fimbria-fornix lesions, or animals given systemic NMDA antagonist [3-(2-carboxypiperazine-4-yl) propyl-1-phosphate] (CPP) show impaired spatial acquisition and memory. Furthermore, real time reverse-transcriptase polymerase chain reaction (RT-PCR) performed on cDNA from hippocampi of trained rats reveal a significant up-regulation of immediate early learning genes 30 minutes after training. These data reflect that this novel paradigm requires the hippocampus for successful spatial acquisition and long-term memory consolidation, and also elicits hippocampal genes previously implicated in learning.

## Methods

Three groups of Long Evans male rats (325-350 g upon arrival, Charles River Laboratories, St-Constant, Qc, Canada) were included in this study: group 1 animals were trained in the spatial paradigm [two separate groups (n = 14) and (n = 5)], group 2 animals trained in a similar, but hippocampus-independent cue paradigm (n = 10), and group 3 animals were behaviorally naive (n = 10). Group 1 rats trained in the spatial paradigm included normal rats, pharmacologic controls (animals given CPP) and surgical controls (fornix-lesioned rats).

### Animals

All rats were housed in pairs, given ad libitum food and water, and kept on a 12 hour light/dark cycle. Rats were allowed to acclimatize to the colony room for at least two days prior to handling. Each rat was handled for two consecutive days before training, injections or surgery. Behavioral training took place in a 180 cm diameter, 80 cm deep pool filled with 60 cm of water (22°C) rendered opaque by the addition of white, non-toxic paint. A video camera mounted to the ceiling directly above the pool recorded swim paths and was connected to a personal computer. Visual cues surrounding the pool included a black and white poster, a neon Frisbee, a hanging plant in a corner and a black curtain. A 21 by 21 cm Plexiglas platform was submerged 3 cm below the water surface in the goal quadrant of the pool, and was made visible by an iron cone that protruded above the water (10 cm). Tracker software (Montreal, Qc) calculated and recorded the latency to platform (sec), and search pathways for each subject on each training trial and probe.

### CPP delivery

#### Pre-training

Rats are adversely affected by i.p. injections of competitive NMDA antagonists with side effects such as hyperactivity, stereotypy, ataxia, and catalepsy [29]. To reduce these behavioral confounds, rats were pre-trained with a full dose of CPP (12 mg/kg, i.p.) one day prior to testing to acclimatize them to the drug. During pretreatment, rats were injected with the drug and returned to their cages for one hour for the drug to take full effect. The rats were then brought to a separate room in which a Tupperware storage bin was filled with warm (25-27°C) water. Individual rats were lowered into the warm water in the container and allowed to swim for 1.5 minutes. Each rat was handled in the water for 10 seconds to provide a short rest, and then allowed to swim an additional 1.5 minutes.

#### Testing

On the actual test day, rats were given i.p. injections of CPP (12 mg/kg), returned to their home cages in the colony room, and one hour later underwent behavioral training.

### Fimbria-fornix surgery

In the surgical control groups, lesions were made to the fimbria-fornix by radiofrequency using Grass Instrument LM4 Lesion Maker. Rats were nothing per os (NPO) 24 hours prior surgery, and were given subcutaneous injections of the anesthetic Acepromazine (0.5 mg/kg) 20 minutes prior surgery. Fifteen minutes prior to placement in the stereotaxic apparatus, the rats were given an intramuscular injection of the anesthetic Ketamine (50 mg/kg), and 5 minutes later, were given a contralateral intramuscular injection of the analgesic Xylazine (5 mg/kg). Rats were also given a subcutaneous injection of atropine sulfate (0.5 mg/kg) to reduce pulmonary secretions.

Measures such as tail and toe pinching were taken to ensure the rat was unconscious prior to placement in the stereotaxic apparatus. After cleaning the skin on the head with iodine, a single anterior-posterior incision was made. Measurements from Bregma were taken: -1.5 AP, +/- 0.8, +/- 2.2 LM, +/- 4.8 DV. Holes were drilled through the skull with a standard, hand-held drill. 8mA or 17 V was delivered for 40 seconds to the lateral positions, while 10 mA or 21 V was delivered for 40 seconds to the medial positions. Antibiotic jelly was applied to the open wound, which was subsequently closed with surgical staples. After surgery, rats were prophylactically treated for infection with intramuscular injections of penicillin (0.1 mL), and treated for pain with a subcutaneous injection of Dipyrone (0.05 mL). Rats were given a 15-day recovery period in the animal facility prior to water maze training. All animal experimentation was conducted according to the guidelines of the Canadian Council on Animal Care and was approved by the Institutional Animal Care Committee.

Multiple studies using fimbria-fornix lesion protocols to impair hippocampus-dependent learning in rats have also assessed the performance of sham-lesioned animals in those tasks. Sham-lesioned rats underwent anesthesia, had cranial lesions made at the same coordinates from Bregma as fimbria-fornix lesioned animals, had insertion of electrodes at those sites, but without current delivery. These sham animals showed no learning impairment in hippocampus-dependent tasks [10,30] and based on these results, no sham-controlled animals were used in this study.

### Brain harvesting and RNA extraction

Immediately following the probe test, each rat trained in either the spatial or cued task was anaesthetized with an overdose intraperitoneal (i.p.) injection of 30% chloral hydrate. Additionally, two (n = 2) rats were handled for two consecutive days, and allowed to swim freely in the water maze pool for 3 minutes, which is the average time to train animals in the spatial paradigm. These 'swim' control rats were injected with 30% chloral hydrate 30 minutes following the exposure to the pool environment and stresses induced by swimming. All rats were decapitated with an animal guillotine, and both hemispheres of hippocampi were isolated and preserved in RNALater (Ambion, Austin, TX).

Learning in spatially trained animals was assessed as described above, and six (n = 6) rats were selected as acceptable spatial learners. One hemisphere of hippocampus per rat was homogenized in RLT Buffer (Qiagen, Alameda, CA) with a hand-held pestle, and run through Qia Shredder columns (Qiagen). RNA was isolated according to Qiagen RNAeasy Mini Kit instructions with minor alterations. RNA was suspended in RNAse-free DEPC-treated water, quantified by spectrophotometry, and stored at -80°C.

### cDNA preparation and RT-PCR

1 μg of RNA per rat hippocampus was reverse-transcribed to cDNA by Moloney Murine Leukemia Virus (M-MLV) Reverse Transcriptase (Invitrogen, Mississauga, ON), according to Invitrogen RT instructions. Total RNA was primed with 5 μg Oligo dT_12-18 _in a total 20 μL reaction volume of 10 mM dNTP mix (10 mM dATP, dGTP, dCTP, and dTTP each, neutral pH), 0.1 M DTT and 5 × First-strand buffer. The reaction was incubated at 65°C for 5 minutes, 37°C for 50 minutes, and finally 70°C for 15 minutes to arrest enzymatic activity.

cDNA from spatially trained and swim-control animals was diluted 1:2 in RNAse/DNAse-free DEPC-treated water. Primer pairs were designed using Primer Express 2.0 (ABI Prism, CA, USA) to amplify gene products 70-151 base pairs long, and were synthesized by Alpha DNA (QC, Canada). Primers were synthesized as follows: *Housekeeping protein p31 *(Accession number BC059141.1): forward tcaaccccaccgtgttcttc, reverse gaggaacccttatagccaaatcc; *Homer1a *(Accession number AJ276327.1): forward cgcaggagaagatggaactga, reverse tttctggtgttaaaggagactgaaga; *c-fos *(Accession number X06769.1: forward tggagccggtcaagaacatt, reverse gccggaaacaagaagtcatca, *zif268 *(Accession number NM_012551.2): forward cagtggccttgtgagcatga, reverse gcagaggaagacgatgaagca. cDNA from one hemisphere of an additional rat trained in the spatial paradigm was used to create a standard curve for real-time RT-PCR. The following quantites of cDNA were used in the standard curve: 2, 1, 1:5, 1:20, 1:40, 1:60, 1:80, 1:100 μL cDNA per sample.

One master mix, created per PCR run, constituted 16 reaction tubes: 6 for each spatially trained rats, 2 for each cage control rat and 8 for each environmental control dilution. PCR reactions were prepared with SYBR Green PCR Core reagents (ABI, CA, USA), and each 25 μL reaction contained 0.624U of AmpliTaq Gold polymerase, 0.25U of AmpErase, 5 mM dNTP mix, 3 mM MgCl_2_, 148 μM 10 × SYBR green buffer, 0.9 mM each primer, and 0.5 μL cDNA.

Primers were designed for the default settings for 3-step PCR using ABI prism 7000 (ABI, CA, USA). 3-step PCR reactions began with a 2 minute incubation period at 50°C, followed by a 10 minute incubation period at 95°C. The third step, which was repeated 40 times, included a 15 second incubation at 95°C, followed by 60 seconds at 60°C. Melt curve analysis began at 60°C and fluorescence was measured to 95°C at an interval of 1.6°C/sec. All PCR products showed one dissociation peak of fluorescence as calculated by the ABI Sequence Detection Software, version 1.1, reflecting a single gene product. Each gene product was further confirmed by electrophoresis on a 2% agarose gel.

A standard curve of the additional spatially trained animal dilutions was calculated by ABI prism software V1.1 (ABI, CA, USA). Threshold cycles (C_T_) [or the first cycle in which fluorescence exceeds a set value above background] for each dilution was calculated and related to the input cDNA concentration. Correlation coefficients were calculated for each standard curve, and each run was only considered if equal to or greater than 0.97. C_T _values were measured for each unknown sample, and quantities of transcript were calculated by the software off the standard curve. Efficiencies of reactions were assessed by slopes of standard curves between candidate genes and *Housekeeping protein p31 *to determine equal quality of amplification between runs prior to normalization. PCR on each gene was repeated in triplicate, and all gene transcript quantities were normalized to the housekeeping gene *Housekeeping protein p31 *transcript quantity.

## Results

### Maze procedures

#### Group 1 - spatial training

**Figure 1:**
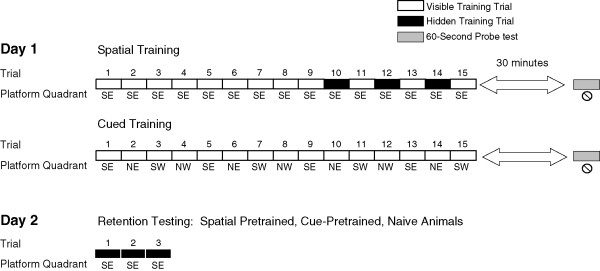
**Paradigms used in behavior training**. The place/cue group was trained with the spatial paradigm: a total of 12 visible and 3 hidden training trials, in which the platform remained fixed in the SE (goal) quadrant of the pool, and a probe test was administered 30 minutes following training. The curtain group had 15 visible training trials in which distal cues were hidden, the platform was rotated in the pool and a probe test was given 30 minutes following training. To assess retention on day 2, spatially pretrained, cued pretrained and naïve animals were given 3 hidden training trials in which the platform remained in the SE goal quadrant.

The spatial paradigm included 15 trials, each separated by a 10 sec inter-trial interval. On each trial, the rat was held by the shoulders, gently lowered into the water facing one of 16 locations along the wall of the pool, and given 60 seconds to find a hidden platform in the SE (goal) quadrant of the pool. If rats failed to locate the platform, they were guided to it by hand. The platform was made visible by a protruding flag on the first 9 consecutive trials, and on the 10th trial, the flag was removed so that the platform was no longer visible. The platform was made alternatively visible and hidden during trials 11 through 15 by the consecutive replacement and removal of the flag. Thirty minutes following training, a probe test was administered in which the platform was removed from the pool, and each rat was allowed to swim freely for 60 seconds (Figure 1).

**Figure 2:**
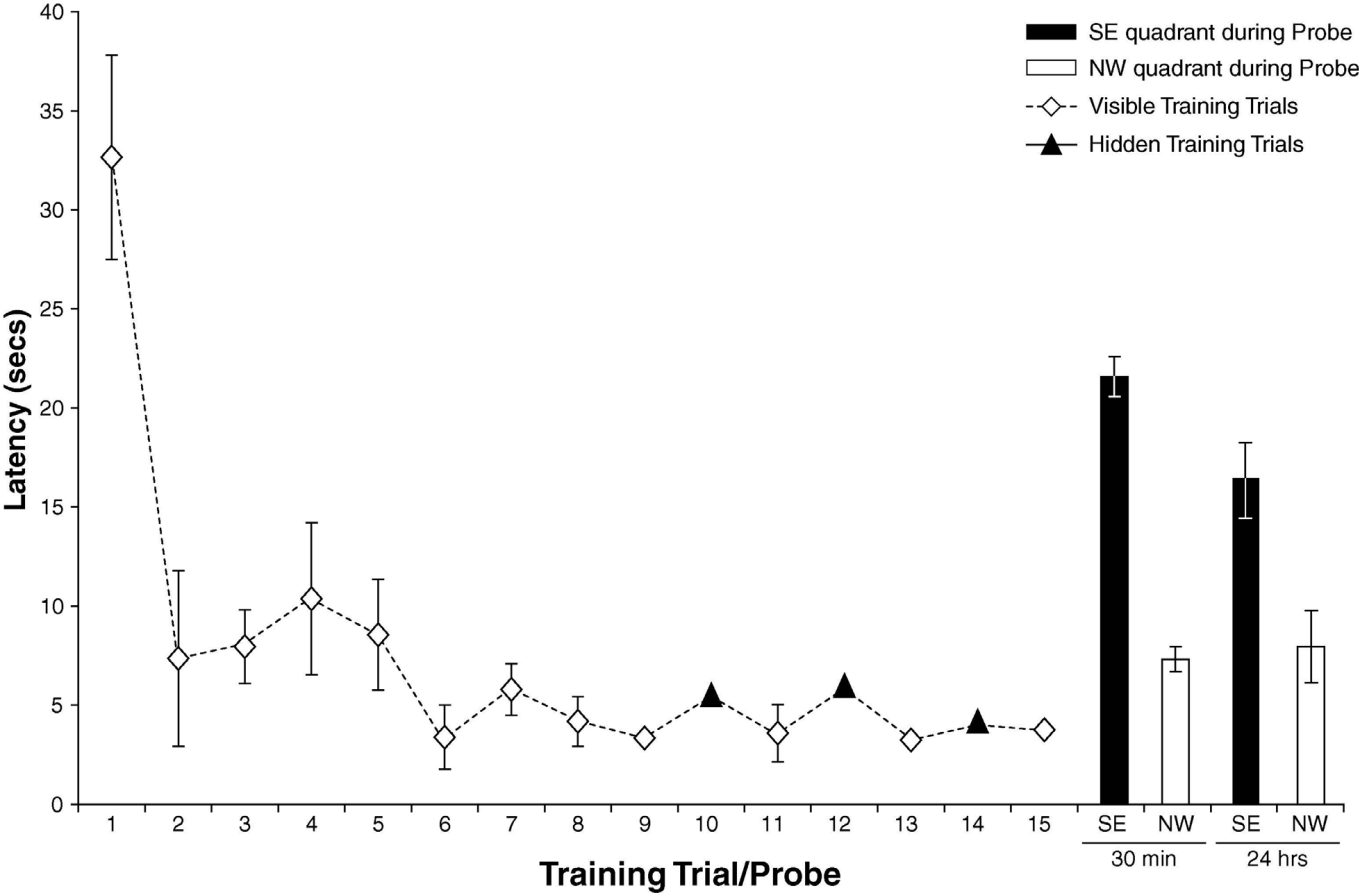
**Latency to platform during spatial training**. Animals (n = 14) were massed trained with 9 cued trials followed by alternation between spatial (trials 10,12,14) and cued trials (11,13,15). Animals show clear improvement across trials 1 to 15, and a bias for the SE goal quadrant, relative to the opposite NW quadrant, during probe testing 30 minutes and 24 hours following training.

All rats in the spatially trained group learned the cued aspect of the task and rapidly approached the visible platform (Figure 2). Seventy percent (14/20) of rats also learned the spatial component of the task and directly approached the training quadrant during the probe test. The rats learned to escape onto the platform rapidly as revealed by a significant decline in escape latency between trial 1 and trial 15 (t (13) = 6.51, p < 0.0001). Performance on hidden and cued trials was equivalent during the last 6 training trials (F (5,8) = 1.70, p > 0.1) indicating that the rats learned to escape equally well onto a visible or hidden platform (Figure 2). Spatial learning was confirmed by the finding that rats spent significantly more time in the SE goal quadrant relative to the opposite, NW quadrant on a probe test given 30 minutes following training (t(26) = 12.11, p < 0.0001) [Figure 2]. During the probe test, spatial learners showed a significantly more time in the goal quadrant then would be expected by 25% chance, or 15 seconds in each quadrant 2-tailed (t(13) = 6.60, p < 0.0001) [Table 1].

**Table 1:** Time Spent in Goal and Opposite Quadrants during Probe Test

	Goal Q (secs)	Opposite Q (secs)	P-value	GQ/15	OQ/15	P-value
**Spatial 30 min**	21.57	7.31	0.0000	1.44	0.49	0.0000

**Spatial 24 hrs**	16.37	8.02	0.0122	1.24	0.38	0.0122

**CPP 30 min**	15.54	15.66	0.9592	1.04	1.04	0.9478

**Fornix 30 min**	6.65	15.55	0.0009	0.44	1.04	0.0007

Data is shown as time spent in goal (training) and opposite quadrants during probe testing in seconds, and as compared to time spent in each quadrant by chance (25% or 15 secs). 2-tailed Student's t-test compare these values between goal (SE) and opposite (NW) quadrants.

#### Group 2 - cued training

**Figure 3:**
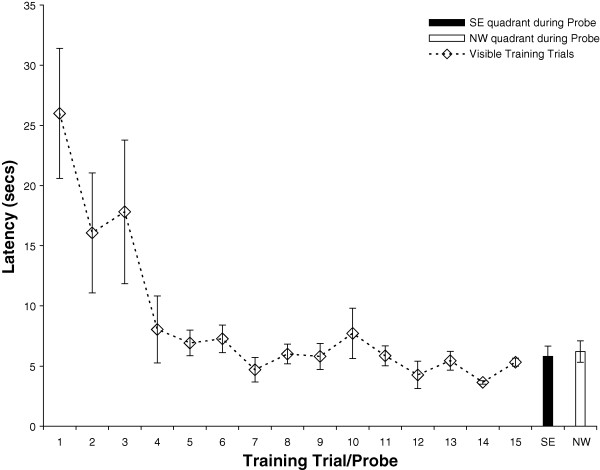
**Latency to platform during cued training**. Animals (n = 10) were trained in the cued training paradigm, in which all training trials (1-15) remained visibly marked. Animals learned to find the visible platform, as demonstrated by decreased latency to platform values from trial 1 to 15. Rats did not use spatial information to locate the platform, as indicated by no preference for the SE goal or NW opposite quadrant during probe testing.

A second cohort of animals was trained in a cued platform paradigm, whereby animals used a hippocampus-independent strategy to find the visible platform in the pool. In order to minimize spatial learning in this task, white plastic shower curtains hid all distal cues surrounding the pool at all times during training and testing. Ten rats (n = 10) were trained with 15 trials in which the platform was made visible by a flag, and was alternated between the SE, NE, SW, NW quadrants of the pool (Figure 1). Rats in the cued group learned the cue-approach task rapidly, and showed a significant improvement in mean latency from the last training trial, trial 15 as compared to trial 1 (t (18) = 5.28, p < 0.0001) (Figure 3). In contrast to the spatially trained group, the curtain group showed no place learning during the probe test (t (18) = 1.22, p > 0.2). Thus, the curtain blocked access to the distal cues, and prevented the rats from learning any spatial location of the platform. These results indicate that access to distal cues was both necessary and sufficient for acquiring spatial memory, and blocking access to those distal cues prevented place, but not cue-approach learning.

#### Memory testing

In order to determine that our version of the Morris water maze elicits long-term memory, two tests were administered to rats 24 hours following initial training. The first assessment examined the performance of five rats trained in the spatial paradigm and given a probe test 24 hours following training. During this probe test, these animals showed significant savings for the goal quadrant relative to the opposite quadrant (t(5) = 2.523, p = 0.0265), and when these latencies were divided by the expected time of 15 seconds (24 hours Q1/15 vs Q3/15 = 2-tailed = t(9) = 3.13, p = 0.012).

**Figure 4:**
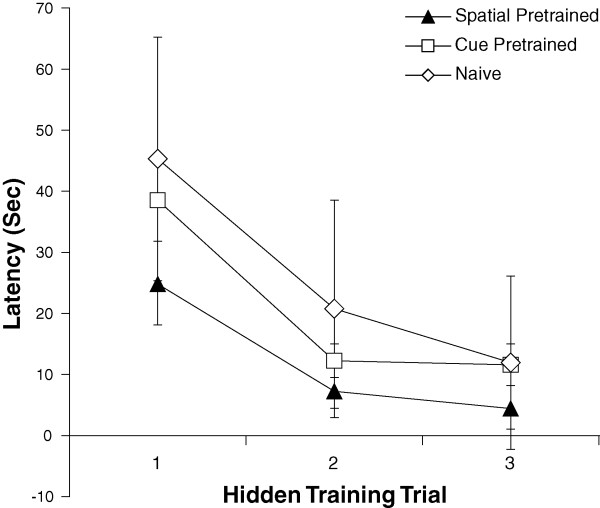
**Latency to platform during retention training and probe test**. Data are shown for rats pretrained in the spatial version of the task (Figure 2), rats pretrained in the cued paradigm (Figure 3), and naïve rats (n = 10). Retention testing took place 24 hours following initial training, and consisted of three hidden training trials. Group differences in performance for trials 1 and 2 show that previous spatial or cue training improves performance one day following training, and that spatial pretraining improves performance better than cue training.

To further assess long-term savings following training, three groups of Long Evans male rats were included in the study: animals trained in the spatial paradigm (n = 20), animals trained in the cued training paradigm (n = 10), and naïve animals, or rats given no behavioral pretraining (n = 10). Spatially trained animals were selected based on their performance on the spatial test given on day 1 of training, and only spatial learners were subsequently trained and tested on day 2 (Figure 1). 24 hours after the initial training, rats in the spatially-trained (n = 14), cue-trained (n = 10), and naïve groups (n = 10) were given 3 training trials in which the platform remained hidden in the SE quadrant (Figure 1, day 2). The mean latency to reach the platform was calculated for the three groups across hidden trials (Figure 4). The spatially-trained group showed significant savings and performed better than either the cue-trained or naïve groups during the first two trials (ANOVA Trial 1 (F(2,31) = 3.49, p < 0.05); Trial 2 (F(2,31 = 4.35, p < 0.05); Trial 3 (F(2,31) = 2.09, p > 0.1). The spatially-trained group performed better on the first retention trial than those given cued training, or naïve animals (escape latency by groups: F (2,31) = 3.49, p < 0.05; planned comparison spatially-trained *versus *cue-trained: t (22) = 1.72, p < 0.05; spatially-trained *versus*. naïve: t (22) = 2.65, p < 0.01). The cue-trained and naïve groups performed similarly (t (22) = 0.74 p > = 0.2). Furthermore, rats in the spatially-trained group escaped onto the platform faster than the other groups of animals during training trial 2 (F(2,31) = 4.35, p < 0.05); planned comparisons: spatially-trained *versus *cue-trained t (22) = 1.84, p < 0.05; spatially-trained *versus *naïve t (22) = 2.74, p < 0.01). Performance by the cue-trained and naïve groups did not differ during trial 2 (t (18) = 1.36, p > 0.05). Therefore, the initial performance differences between groups were due to training parameters, not spatial learning abilities.

### Surgical controls: fimbria-fornix lesioned animals

**Figure 5:**
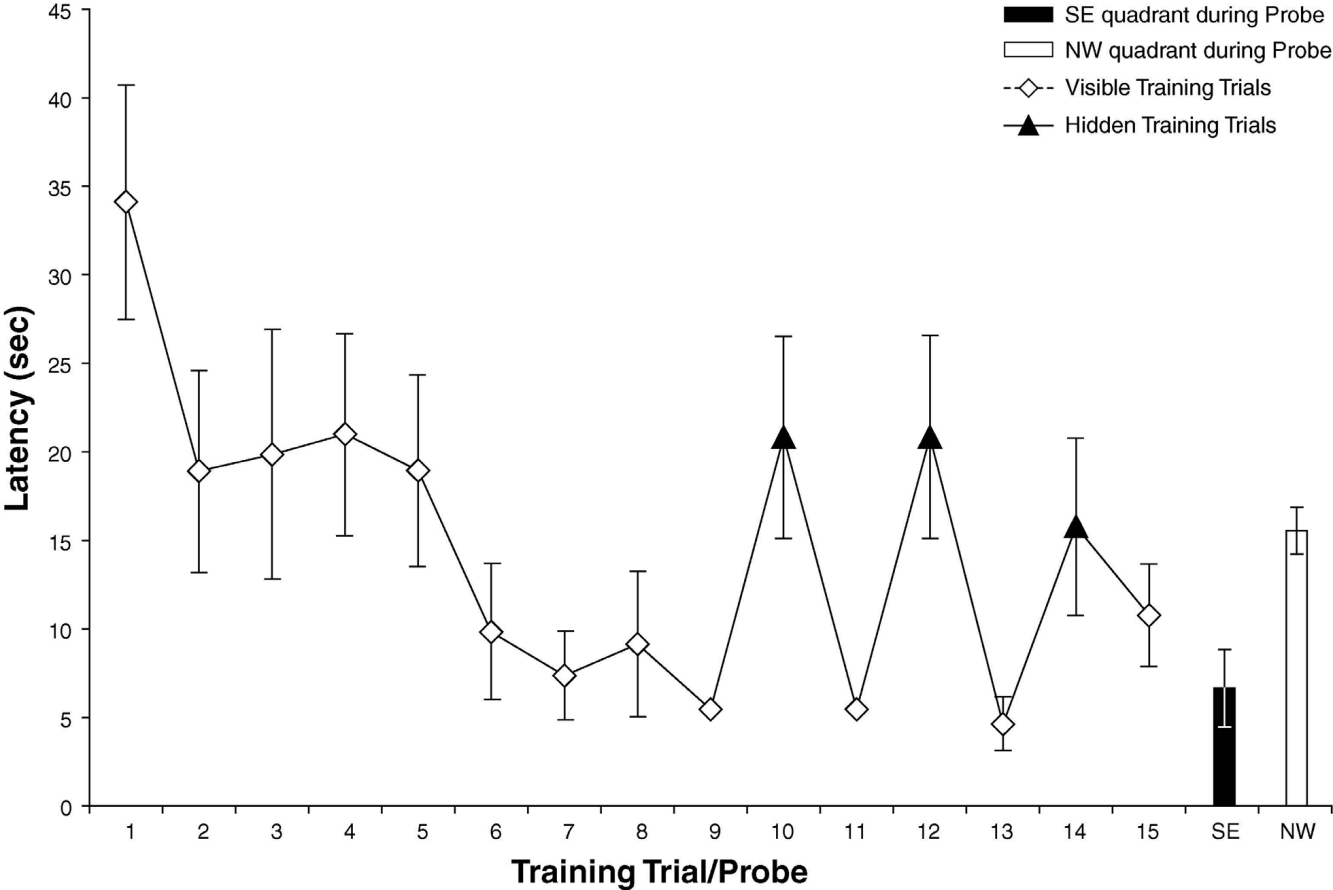
**Fornix-lesioned latency to platform during spatial training**. Animals (n = 12) underwent bilateral fimbria-fornix ablation 15 days prior training, yet still show clear improvement across trials 1 to 15. Animals show no bias for the SE goal quadrant, and spend significantly more time in the opposite NW quadrant during probe testing.

Radiofrequency was used to ablate the fornix in 12 animals, and 15 days post-surgery, these rats were trained in the spatial paradigm (Figure 5). As expected, the lesioned animals performed as well as normal animals across the cued training trials (t(22) = 3.26, P < 0.005), since this performance is not dependent on hippocampal function. However, these animals showed impaired learning on the hidden training trials, as shown by greater latency values during training trials 10, 12, and 14. Furthermore, animals did not show a bias for the SE goal quadrant during probe testing (t(22) = 3.49, p = 0.001 (Figure 5 and Table 1).

### Pharmacological controls: CPP animals

24 hours following pretreatment with CPP, rats were given a second dose of CPP and trained in the spatial paradigm (data not shown). Like the fimbria-fornix lesioned animals, these rats performed as well as normal animals across the cued training trials 1 to 15 (t(22) = 3.57, p < 0.005). While spatially-trained rats show distinct improvement on the hidden trials (trials 10, 12, 14), the CPP-treated rats performed poorly on the hippocampus-dependent trials. Furthermore, CPP-treated animals did not show a bias for the SE goal quadrant during probe testing (Table 1).

### RT-PCR

**Figure 6:**
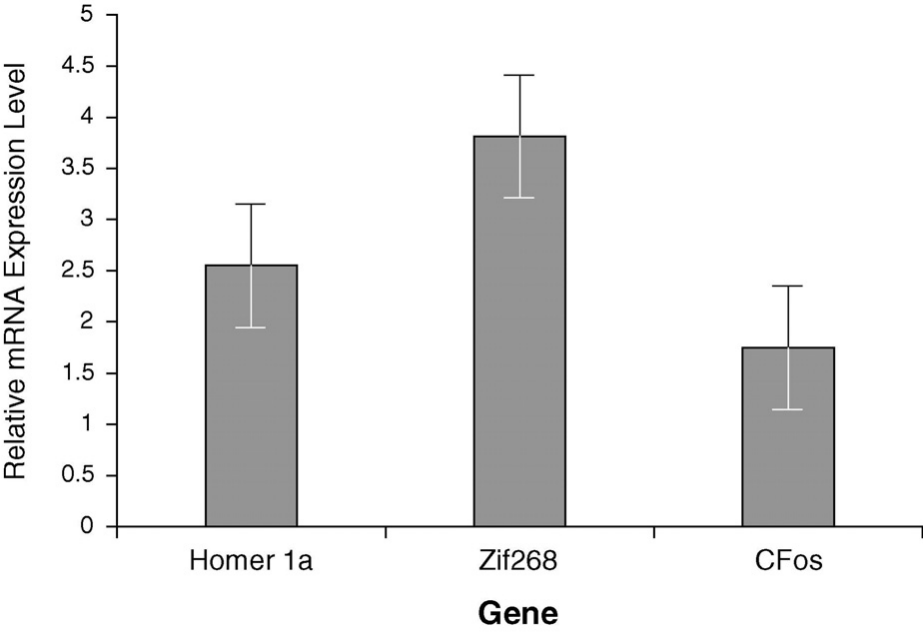
**Differential mRNA expression as a function of spatial behavior**. Changes in level of transcript were determined by real-time RT-PCR as described in Materials and Methods. Compared to swim control animals (n = 2), spatially trained rats (n = 6) had higher levels of gene transcript in the hippocampus 30 minutes following training in the spatial paradigm. [PCR CT values: homer1a (t(21) = 6.07, p < 0.0001); c-fos (t(22) = 7.121, p < 0.0001); zif268 (t(22) = 7.601, p < 0.0001)]. Fold-changes: homer1a = 2.6, zif268 = 3.8, c-fos = 1.8.

To determine gene expression as a function of spatial training, real time PCR was used to measure transcript levels of selected immediate early genes previously associated with learning, *Homer1a, zif268 *and *c-fos*, thirty minutes following spatial learning compared to naïve animals. Rats were trained with 15 training trials as described above, returned to their home cages for 30 minutes, given a 60 second probe test, and promptly sacrificed. RNA was extracted from one hemisphere of the hippocampus of spatial learners (n = 6) and swim-control animals (n = 2), and RNA was reverse-transcribed to cDNA. Quantitative real-time PCR was performed using this cDNA to measure transcript levels of the three immediate early genes, as well as *Housekeeping protein p31 *which served to normalize the samples. Thirty minutes following spatial training, there was a significant increase in the hippocampal transcript levels of *Homer1a *(2.6 fold), *c-fos *(1.8 fold), and *zif268 *(3.8 fold) relative to swim-control rats (Figure 6).

## Discussion

Animals trained in our spatial paradigm perform similarly to reported animals trained in multiple-day Morris water maze tasks. Our spatially-trained animals show steady improvement across training trials, as reflected by decreased latency to mount the platform, regardless whether the platform is hidden or visible. In contrast, animals that underwent fimbria-fornix lesions, or given systemic NMDA antagonist drug display similar improvement across visible training trials, but show profound deficit on the hidden training trials. While spatially-trained animals spend more time in the goal quadrant during probe testing, the lesioned and CPP animals do not show this bias, indicating poor spatial acquisition. These data are consistent with the literature showing that fimbria-fornix lesions [10,31] and administration of CPP [32,33] impair the performance on standard Morris water maze tasks in similar ways.

Animals trained in our abbreviated Morris water maze task generate lasting long-term spatial memory, as demonstrated by their preference for the goal quadrant during probe testing 24 hours following training (Table 1). Furthermore, spatially trained animals show long-term memory on retention testing, as compared to cue-trained and naïve animals. Of the three cohorts of rats, only rats previously trained in the spatial paradigm showed long-term memory for the location of the platform during the first trial of training during retention testing as shown in Figure 4. The retention test alone served as a massed spatial training paradigm: by the third trial, there was no significant difference between the latencies of naïve, cued-pretrained, and spatially-pretrained animals. Interestingly, pretraining in the cued version of the task did not offer any benefit to subsequent spatial learning, since there were no significant differences in latencies between cued-pretrained and naïve animals during any of the trials. These results resemble the data collected from normal and Alzheimer transgenic mice, in which 24 hours after training in a water task with visible platforms, mice were given three hidden trials in which the platform remained in the same goal location. While both groups of animals performed equally well on visible training trials the day earlier, only normal mice showed a reduced latency on the first hidden training trial. The transgenic mice, however, learned to find the hidden platform on trials 2 and 3, indicating the three hidden training trials served as a new learning experience [34].

The training paradigm introduced here offers unique features that are not utilized in other massed versions of water maze tasks. We use nine consecutive visible training trials which give animals ample time to equilibrate to the training room, the cool water, and the physical practice of swimming. These trials also provide the opportunity for rats to attenuate their stress and avoid behavioral despair by appreciating the existence of an escape platform, and to overcome their natural tendency to remain on the periphery of the pool. These training trials do not require hippocampal function, as surgical and pharmacological controls show similar performance as normal rats on these trials. As such, these trials serve as an internal control for each animal and allow researchers to identify the baseline visual, motor, stress and motivational capabilities of their subjects. We also show that nine trials are sufficient to allow the rodent to overcome its natural tendencies of thigmotaxis, and to use goal-directed behavior to escape on the platform.

The hidden trials (trials 10, 12, 14) are used to train the animals to continue to search for a platform despite it not being visible, and serve as a preliminary assessment of spatial learning. Clearly successful performance during these trials requires an intact and functioning hippocampus, as shown by the increased latencies in the surgical and pharmacological controls on these trials. By interspersing the hidden trials amongst the visible trials, we encourage animals to alter their acquisition from cue-approach learning to spatial navigation. The probe test given 30 minutes following training serves as a second and more definitive measure of spatial learning.

Making the transition from cue-approach to spatial learning is sensitive enough to distinguish the performances of fimbria-fornix lesioned animals and NMDA-antagonized animals from normal spatial learning rats. However, this transition is not simple for rats on a whole, since only 70% of normal rats will transition well. The relative difficulty of this transition, however, can likely be used to assess subtle differences in cognitive abilities between animal cohorts. Whereas multiple day training might provide the opportunity to overcome mild learning and memory deficits with over-training, this more challenging version we present here may be sensitive enough to show these deficits. The caveat with having baseline success around 70% is that researchers will require increased number of animals per cohort to properly assess overall learning abilities.

The Morris water maze is used to distinguish cognitive abilities between a large variety of experimental rodent models based on pharmacological manipulation, brain trauma, toxic exposure and aging, to name a few. Some rodents within these experimental cohorts are faced with unavoidable co-morbidities that may lead to fatigability, motor impairment, or increased susceptibility to anxiety and behavioral despair. Any of these will make training in the original multiple-day Morris water maze task difficult. Performance on the visible training trials will allow detection of these abnormalities, and reflects each individual baseline level. Since the entire training period takes less than 15 minutes, animals that might be impaired by the physical exhaustion of longer training, may be good candidates for our abbreviated paradigm. Furthermore, the brevity in training allows researchers to more closely identify the time frame in which acquisition took place, which is difficult, if not impossible, to ascertain with multiple-day Morris water maze tasks.

## Limitations

The challenges in implementing this abbreviated water maze task are those that are inherent to any massed training paradigm. Approximately 70% of normal rats are able to successfully learn the paradigm, and therefore larger cohorts of rats are required to overcome the relative difficulty of the task. As with any maze paradigm, successful training requires normal perception, physical strength and coordination, as well as appropriate stress and motivational responses. Hence experimental subjects and controls should be carefully screened to exclude physical and emotional disabilities. Finally, this task might be too physically demanding for mice, and therefore should be modified in order to accommodate mouse size and physical strength.

## Conclusions

Our paradigm uses visible training trials to serve as a baseline control measure and hidden training trials as a preliminary assessment of spatial learning. A probe test given 30 minutes after training confirms spatial learning, and short-term memory for the platform location, and a probe test administered 24 hours after training confirms lasting long-term spatial memory is generated. This paradigm gives the opportunity to dissociate poor performance along these measures (cue approach acquisition, spatial acquisition, and short-term and long-term spatial memory), which can allow accurate study of animal deficit. It is a sensitive enough measure to detect hippocampal learning deficits from surgically and pharmacologically impaired animals, and elicits hippocampal expression of genes that are established as important to learning. Hence, we introduce this novel paradigm as a useful tool to quickly and efficiently discern learning disabilities.

## Competing interests

The authors declare that they have no competing interests.

## Authors' contributions

LAF participated in the design of the paradigm, performed the fimbria-fornix ablation surgeries, tested the animals, carried out RT-PCRs, performed statistical data analysis and drafted the manuscript. MLS participated in the design of the paradigm, organized the appropriate control cohorts, oversaw the statistical analysis of the behavior data, and helped to draft the manuscript. JN conceived of the study and participated in its design and coordination and helped to draft the manuscript. All authors have read and approved the final manuscript.
